# The reverse transsulfuration pathway affects the colonic microbiota and contributes to colitis in mice

**DOI:** 10.1007/s00726-024-03423-4

**Published:** 2024-10-19

**Authors:** Alain P. Gobert, Yvonne L. Latour, Kara M. McNamara, Caroline V. Hawkins, Kamery J. Williams, Mohammad Asim, Daniel P. Barry, Margaret M. Allaman, Alberto G. Delgado, Ginger L. Milne, Shilin Zhao, M. Blanca Piazuelo, M. Kay Washington, Lori A. Coburn, Keith T. Wilson

**Affiliations:** 1 Division of Gastroenterology, Hepatology, and Nutrition, Department of Medicine, Vanderbilt University Medical Center, Nashville, TN 37232 USA; 2 Center for Mucosal Inflammation and Cancer, Vanderbilt University Medical Center, Nashville, TN 37232 USA; 3 Program in Cancer Biology, Vanderbilt University Medical Center, Nashville, TN 37232 USA; 4 Department of Pathology, Microbiology, and Immunology, Vanderbilt University Medical Center, Nashville, TN 37232 USA; 5 Department of Biostatistics, Vanderbilt University Medical Center, Nashville, TN 37232 USA; 6 Veterans Affairs Tennessee Valley Healthcare System, Nashville, TN 37232 USA

**Keywords:** Cystathionine g-lyase, Inflammatory bowel disease, Colitis, Colitis-associated carcinogenesis, Cysteine, Gut microbiota

## Abstract

Cystathionine γ-lyase (CTH) is a critical enzyme in the reverse transsulfuration pathway, the major route for the metabolism of sulfur-containing amino acids, notably converting cystathionine to cysteine. We reported that CTH supports gastritis induced by the pathogen *Helicobacter pylori*. Herein our aim was to investigate the role of CTH in colonic inflammation. First, we found that CTH is induced in the colon mucosa in mice with dextran sulfate sodium-induced colitis. Expression of CTH was completely absent in the colon of *Cth*^–/–^ mice. We observed that clinical and histological parameters are ameliorated in *Cth*-deficient mice compared to wild-type animals. However, *Cth* deletion had no effect on tumorigenesis and the level of dysplasia in mice treated with azoxymethane-DSS, as a reliable model of colitis-associated carcinogenesis. Mechanistically, we determined that the deletion of the gene *Slc7a11* encoding for solute carrier family 7 member 11, the transporter of the anionic form of cysteine, does not affect DSS colitis. Lastly, we found that the richness and diversity of the fecal microbiota were significantly increased in *Cth*^–/–^ mice compared to both WT and *Slc7a11*^–/–^ mice. In conclusion, our data suggest that the enzyme CTH represents a target for clinical intervention in patients with inflammatory bowel disease, potentially by beneficially reshaping the composition of the gut microbiota.

## Introduction

In mammals, the major route for the metabolism of sulfur-containing amino acids occurs through the reverse transsulfuration pathway (RTP) via the action of cystathionine b-synthase (CBS) and cystathionine g-lyase (CTH) (Aitken and Kirsch 2005; Chiku et al. 2009; Singh et al. 2009). These cytosolic enzymes utilize pyridoxal 5′-phosphate as a cofactor and homocysteine as a first substrate to generate cystathionine. More specifically, CBS catalyzes the condensation of homocysteine with serine to form cystathionine, whereas CTH condenses cysteine with homocysteine to generate cystathionine and hydrogen sulfide (H_2_S). Cystathionine is then converted into cysteine, a-ketobutyrate, and ammonia by CTH. Of importance, CTH can also generate H_2_S by a, b-elimination of cysteine to generate serine, a, g-elimination of homocysteine to synthesize homoserine, b-replacement of cysteine to form lanthionine, and g-replacement of homocysteine leading to homolanthionine synthesis.

The biological activity of CTH in homeostatic conditions relies principally on *i*) the biosynthesis of the gasotransmitter H_2_S, which supports endothelium-dependent vasorelaxation (Yang et al. 2008), reduces oxygen- and nitrogen-mediated oxidations (Liu et al. 2022), and regulates inflammation (Stummer et al. 2023); and *ii*) generation of the semi-essential amino-acid cysteine, a precursor of the formation of the antioxidant tripeptide glutathione and of the broad-spectrum cytoprotective agent taurine (Huxtable 1992). However, CTH can be induced during pathological conditions and the dysregulation of the RTP may have pathophysiological consequences (Sbodio et al. 2019). Hence, we reported that pathogenic bacteria stimulate the expression of the gene *Cth* in macrophages through a PI3K/MTOR/SP1 signaling pathway (Gobert et al. 2019). Consequently, cystathionine synthesis is increased, thus facilitating survival of pathogens within myeloid cells, and macrophage activation is affected by putrescine accumulation and increased histone methylation (Gobert et al. 2019). Further, *Cth*-deficient mice exhibit reduced mucosal immune response and gastritis during infection with the gastric pathogen *Helicobacter pylori* (Latour et al. 2022). These data support the concept that CTH induction favors the pathogenicity of *H. pylori* and sustains inflammation in the stomach. In this context, the aim of the present paper was to determine the role of CTH in inflammation in the colon and, by extension, in colitis-associated carcinogenesis (CAC).

Herein, we found that *Cth* deletion protects mice from colitis induced by dextran sulfate sodium (DSS). However, tumorigenesis in animals treated with azoxymethane (AOM)-DSS, as a model of CAC, was similar between WT and *Cth*^–/–^ mice. Mechanistically, we determined that mice lacking the gene *Slc7a11*, which encodes for the transporter of the anionic form of cysteine, cystine, namely solute carrier family 7 member 11 (SCL7A11, also termed xCT), are not protected from DSS colitis, suggesting that the RTP-dependent synthesis of cysteine is not involved in the pathophysiology of colitis. We also observed that CTH, but not SLC7A11, dysregulates the intestinal microbiota. Therefore, the RTP represents a potential target for clinical intervention in patients with inflammatory bowel disease (IBD).

## Materials and methods

### Mice and experimental models

Age-matched (8–12 wk) wild-type (WT), *Cth*^–/–^ (Yang et al. 2008), and *Slc7a11*^–/–^ mice (Sato et al. 2005) on the C57BL/6 background were house-bred in our animal facility and were fed 5L0D chow (LabDiet).

For the colitis model, age-matched (8–12 wk old) male mice were treated with 4% DSS (mol. wt. 36,000–50,000; TdB Labs) for 5 days, followed by 5 more days of regular water (Gobert et al. 2018, 2023; Singh et al. 2019). For the CAC model, mice received one intraperitoneal injection of AOM (12.5 mg/kg; Millipore) followed by 3 cycles of 4% DSS for 4 days beginning at day 5, 26, and 47, and were euthanized on day 56 or sooner if moribund (Gobert et al. 2021, 2022, 2023). Mice were weighed daily or weekly for the colitis and CAC model, respectively. At sacrifice, colons were removed, measured, opened longitudinally, washed, weighed, and Swiss-rolled for histology. In the AOM-DSS experiments, tumors were also counted and measured in two dimensions in the distal colon with electronic calipers under a dissecting microscope (Gobert et al. 2021, 2022, 2023). Tumor burden was determined by the sum of the areas of all tumors.

Animals were used under protocol M20000047, which was approved by the Institutional Animal Care and Use Committee at Vanderbilt University, the Vanderbilt University Institutional Biosafety Committee, and the Research and Development Committee of the Veterans Affairs Tennessee Valley Healthcare System. Procedures were performed in accordance with institutional policies, AAALAC guidelines, the AVMA Guidelines on Euthanasia, NIH regulations (Guide for the Care and Use of Laboratory Animals), and the United States Animal Welfare Act (1966).

### Histological analysis

Colons were fixed in formalin and embedded in paraffin. Sections (5 μm) were stained with hematoxylin and eosin (H&E) and examined in a blinded manner by a gastrointestinal pathologist (M.K.W.). The comprehensive histological injury score for DSS colitis involves both inflammatory and epithelial damage parameters: inflammation severity (0–3) and inflammation extent (0–3) were each multiplied by the percent involvement (1 = 0–25%, 2 = 25–50%, 3 = 50–75%, and 4 = 75–100%) and added together to yield the inflammation score (0–24); the parameter of crypt damage (0–4) was also multiplied by the percent involvement to yield an epithelial injury score (0–16). These scores were then added together to yield the histologic injury score (0–40) (Gobert et al. 2018, 2022, 2023). The severity of dysplasia was determined as described for the AOM-DSS model (Gobert et al. 2022, 2023; Hardbower et al. 2017).

### Immunostaining

Immunohistochemistry (IHC) was performed on the Swiss-rolled colons. Sections were deparaffinized and incubated at room temperature with 3% hydrogen peroxide in phosphate-buffered saline to block endogenous peroxidase and blocked for 1 h in Protein Block Serum-Free (Dako). Slides were sequentially incubated with *i*) a rabbit polyclonal anti-CTH antibody (MyBiosource; 1:100) and MACH 2 Rabbit HRP-Polymer (Biocare Medical) or *ii*) a rabbit monoclonal anti-IL-1b antibody (Abcam; 1:500) and with EnVision+/HRP, Rabbit, HRP (Dako). Detection was performed using 3,3′-diaminobenzidine and tissues were counterstained by hematoxylin.

### ELISA

Colon tissues were lysed by the CelLytic MT Cell Lysis Reagent (Sigma) and the protein concentration was determined by Pierce BCA Protein Assay Kit (Thermo Fisher Scientific). The concentration of IL-1b in these lysates was quantified using the Mouse IL-1 beta ELISA Kit (Proteintech).

### Measurement of cysteine concentration

Frozen colon biopsies were homogenized in 0.1 M trichloroacetic acid containing 10^− 2^ M sodium acetate, 10^− 4^ M EDTA, and 10.5% methanol (pH 3.8). After a 10,000 *g* centrifugation for 20 min, the supernatants were used for protein assay using BCA and for LC/MS, as we reported (Gobert et al. 2019).

### Analysis of mRNA expression

Total RNA was isolated using the RNeasy Mini kit (Qiagen). Reverse transcription was then performed using Superscript II Reverse Transcriptase (Invitrogen) and Oligo dT, and mRNAs were amplified using the PowerUp SYBR Green Master Mix (Thermo Fisher Scientific) and the following primers: mouse *Cth*: F: TGCTAAGGCCTTCCTCAAAA, R: GTCCTTCTCAGGCACAGAGG; mouse *Cbs*: F: TCATCCTGCCTGACTCTGTG, R: CAGCTCTTGAACACGCAGAC; mouse *Il1b*: F: ACCTGCTGGTGTGTGACGTTCC, R: GGGTCCGACAGCACGAGGCT; mouse *Il17*: F: ATCCCTCAAAGCTCAGCGTGTC, R: GGGTCTTCATTGCGGTGGAGAG; mouse *Actb*: F: CCAGAGCAAGAGAGGTATCC, R: CTGTGGTGGTGAAGCTGTAG. The relative expression of target genes was calculated by the 2^–DDCt^ method, using *Actb* as the housekeeping gene.

### Fecal microbiota analysis

Mice were euthanized and feces in the colon were harvested, weighed, and genomic DNA was extracted using the QIAamp Fast DNA Stool Mini Kit (Qiagen). DNA was quantified using Qubit Fluorometric Quantification (Thermo Fisher Scientific) and the V4 region of the 16 S rRNA gene was sequenced with the Illumina MiSeq. Sequences were processed with Mothur, version 1.44 (Schloss et al. 2009), aligned to the SILVA database release 132, and taxonomically classified with the Ribosomal Database Project classifier 16 (Cole et al. 2009; Pruesse et al. 2007). Non-bacterial sequences and chimeric sequences detected by UCHIME were removed. Operational Taxonomic Unit clustering was performed with VSEARCH, using abundance-based greedy clustering (Rognes et al. 2016). Rarefaction followed by alpha-diversity, and beta-diversity calculations were repeated 1000 times, and the results were averaged.

The 16 S rRNA sequencing of the fecal microbiota has been deposited on the Sequence Read Archive website with the BioProject ID PRJNA1096754 (Temporary Submission ID: SUB14361309).

### Statistics

Figures were designed and statistics were executed using GraphPad Prism 10.3.1. All the data are expressed as the mean ± SEM. Outliers were identified using the ROUT test (Q = 5%) and removed from the analysis. Data that were not normally distributed according to the D’Agostino & Pearson normality test were log or square root transformed, and distribution was re-assessed. Student’s *t* test or ANOVA with the Tukey test were used to determine significant differences between two or multiple groups, respectively. When data were not normally distributed, a Kruskal-Wallis test followed by a Mann-Whitney U test was performed. Contingency analysis was performed by the Fisher’s exact test.

The relative abundance of the phyla and genus of the intestinal microbiota was analyzed by the Kruskal-Wallis test after multiple comparison adjustment by the Benjamini & Hochberg method. Alpha-diversity was estimated with the inverse Simpson index. A beta-diversity dissimilarity matrix (Bray-Curtis) was computed over the multiple rarefactions and PERMANOVA was used to test for associations between the three groups.

## Results

### Induction of CTH in experimental colitis

**Fig. 1:**
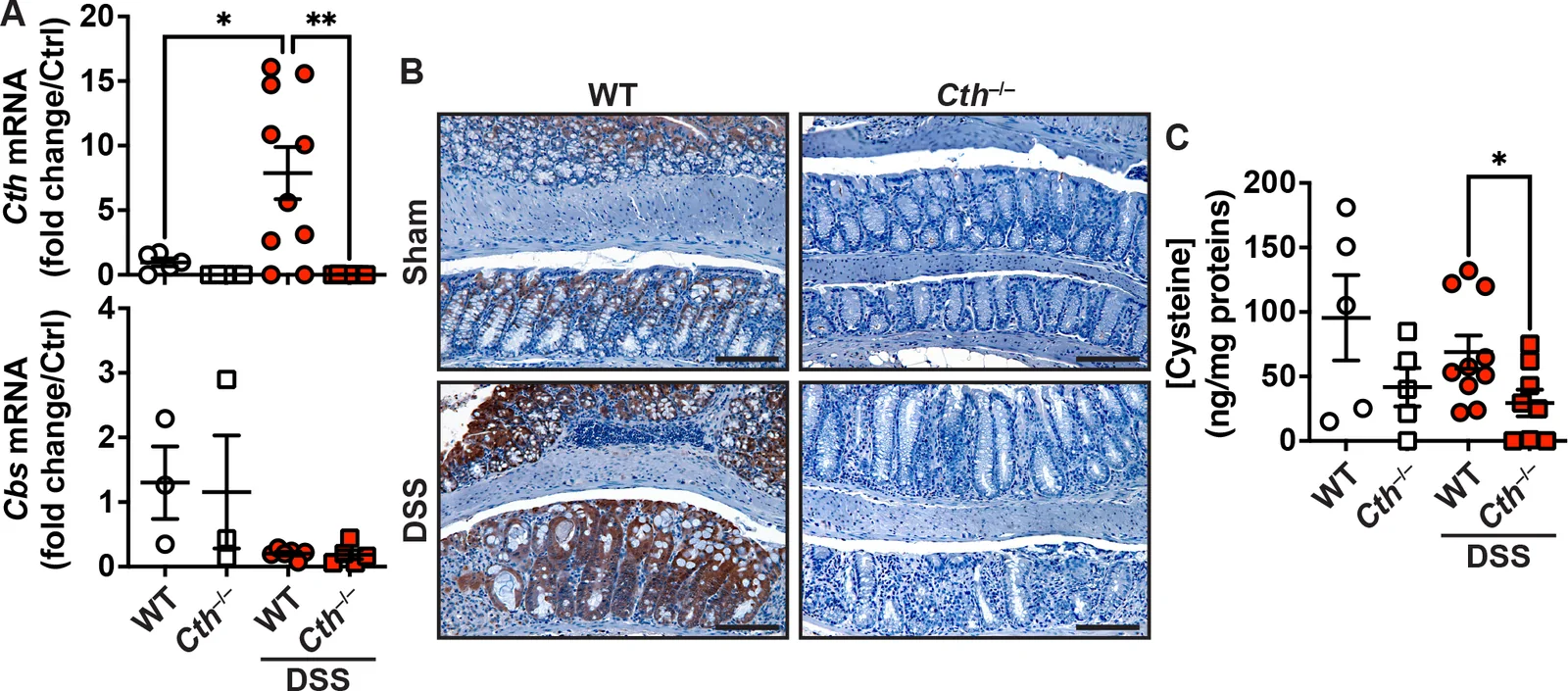
Induction of CTH expression in murine colitis. (**A**) RNA was extracted from colon biopsies from WT or *Cth*^–/–^ mice treated or not with DSS. *Cth* and *Cbs* mRNA levels were determined by RT-real-time PCR and semi-quantitative analysis. (**B**) The colon of these mice was also immunostained for CTH. These are representative images from 5 mice per group; scale bars, 100 μm. (**C**) Cysteine concentration measured by LC/MS. in **A** and **C**, each dot represents a mouse; *P < 0.05, **P < 0.01 by one-way ANOVA and Tukey test

We previously reported that CTH is induced in gastric macrophages from patients with gastric inflammation and cancer (Gobert et al. 2019), as well as mice with *H. pylori*-mediated gastritis (Latour et al. 2022). We now found that expression of the gene *Cth* was significantly induced in the colon of WT mice treated with DSS compared to sham control animals (Fig. 1A). As expected, *Cth* mRNA was not detected in the colon of *Cth*^–/–^ mice that were given DSS or not (Fig. 1A). The expression of the gene *Cbs* that encodes for CBS, the second enzyme of the RTP, was not affected by DSS treatment nor *Cth* deletion (Fig. 1A).

By IHC, we observed that CTH is expressed in colonic epithelial cells (CECs), at the upper part of the crypts in C57BL/6 mice (Fig. 1B). After DSS treatment, the level of CTH immunostaining was markedly increased in CECs and was expanded to the lower part of the crypts (Fig. 1B). Deletion of *Cth* led to complete loss of CTH protein in the colon of mice treated or not with DSS (Fig. 1B).

Although it has been shown that daily food intake is not altered by *Cth* deletion (Ishii et al. 2010), we observed that cysteine, which is biosynthesized through the RTP, was significantly reduced in the colon of *Cth*^–/–^ mice compared to WT mice after DSS treatment (Fig. 1C). *Cth*^–/–^ naïve mice also exhibited less cysteine concentration in the colon, although the difference did not reach statistical significance (Fig. 1C).

### Deletion of ***Cth*** improves DSS colitis

**Fig. 2:**
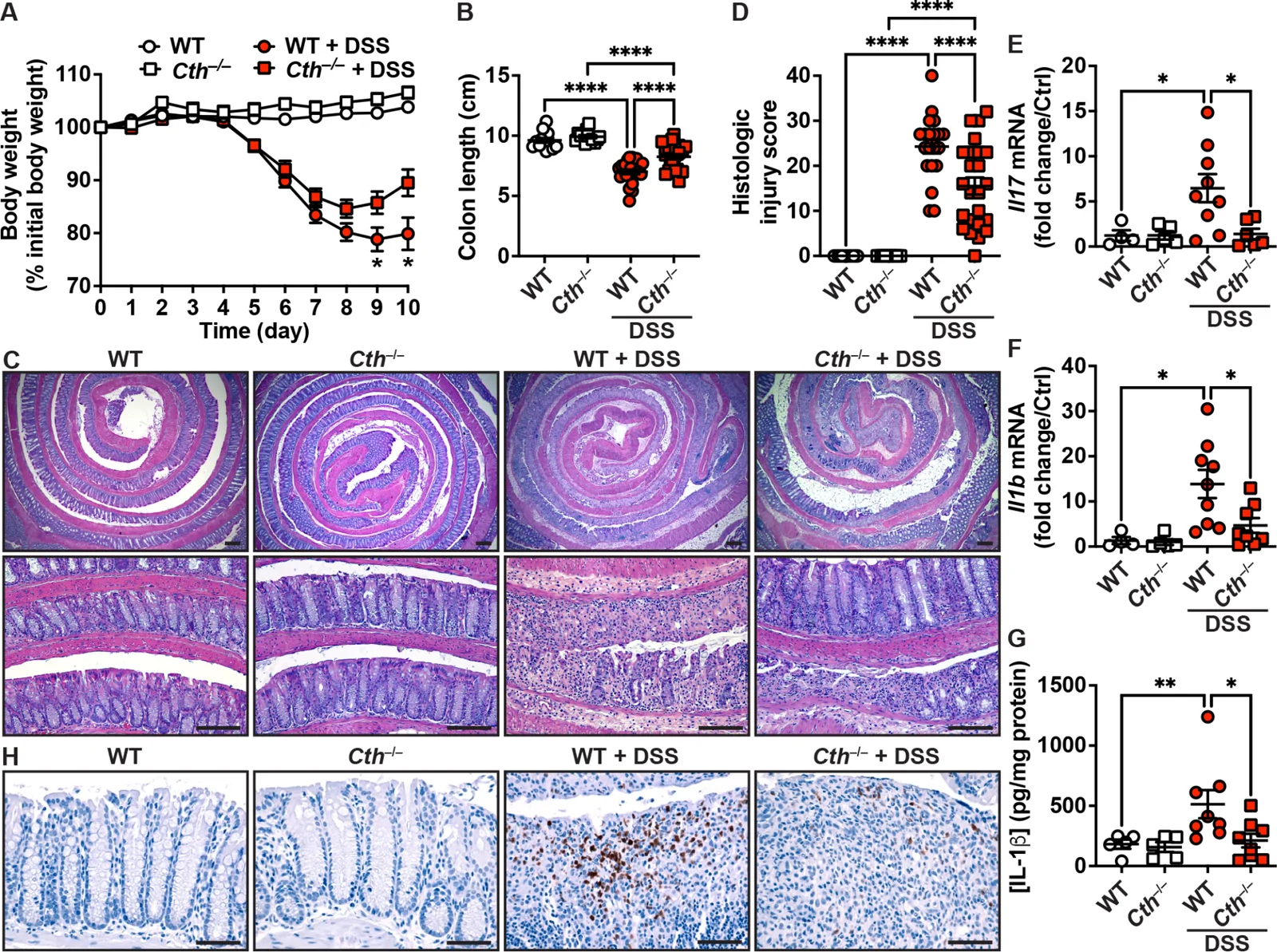
Impact of *Cth* deletion on DSS colitis. WT and *Cth*^–/–^ C57BL/6 mice were treated or not with 4% DSS for 5 days and then kept for 5 more days. (**A**) Body weights were measured daily and are shown as percentage of initial body weight. *P < 0.05 compared to *Cth*^–/–^ + DSS by two-way ANOVA and Tukey test; *n* = 13 untreated mice for both genotypes; *n* = 23 WT + DSS; *n* = 24 *Cth*^–/–^ + DSS. (**B**) The length of the colon was measured. **C-D.** Colons were Swiss-rolled and stained with H&E (**C**) and scored for histologic injury (**D**); scale bars, 100 μm. **E-F.** The expression of *Il17* (**E**) and *Il1b* (**F**) mRNA was analyzed in the colon by RT-real-time PCR. **G.** The concentration of IL-1b was determined by ELISA. **H.** Immunodetection of IL-1b by IHC in the colon. These are representative images of 3 controls and 5 DSS-treated mice per genotype. *P < 0.05, **P < 0.01, ****P < 0.0001 by one-way ANOVA and Tukey test (**B** to **F**) or one-way ANOVA with the Kruskal-Wallis test, followed by the Mann-Whitney *U* test (**G**); each dot represents a mouse

WT and *Cth*^–/–^ mice began losing weight on day 5 after starting DSS (Fig. 2A). However, at day 9 and 10, there was significantly less body weight loss in *Cth*^–/–^ mice compared to WT animals (Fig. 2A). In addition, DSS-induced colon shortening in WT mice was significantly improved in *Cth*-deficient mice (Fig. 2B). In WT mice treated with DSS, we observed epithelial damage, crypt loss, and strong colonic inflammation evidenced by immune cell infiltration in the colonic mucosa (Fig. 2C), consistent with our prior studies (Gobert et al. 2018, 2022; Singh et al. 2019). These histologic injuries were markedly reduced in DSS-treated *Cth*^–/–^ mice (Fig. 2C). Using a comprehensive scoring system quantifying inflammation and epithelial damage, we showed a significant reduction in histologic injury in *Cth*^–/–^ mice compared with WT animals in response to DSS (Fig. 2D). There was no detectable inflammation or epithelial injury in sham-treated WT or *Cth*^–/–^ mice (Fig. 2C and D). Assessment of the mRNA expression of the prototype Th17 gene *Il17* and the innate immune response gene *Il1b* demonstrated a marked increase with DSS colitis in the WT mice that was significantly attenuated in *Cth*^–/–^ mice (Fig. 2E and F). Further, we found by ELISA that IL-1b protein level was increased in the colon of mice that were given DSS; however, the concentration of IL-1b protein was significantly reduced in DSS-treated *Cth*^–/–^ mice compared to WT animals (Fig. 2G). This was confirmed by IHC for IL-1b, which was mainly present in the inflammatory lesions of DSS-treated mice and markedly less abundant in *Cth*-deficient mice (Fig. 2H).

### CTH does not affect inflammation-mediated tumorigenesis

**Fig. 3:**
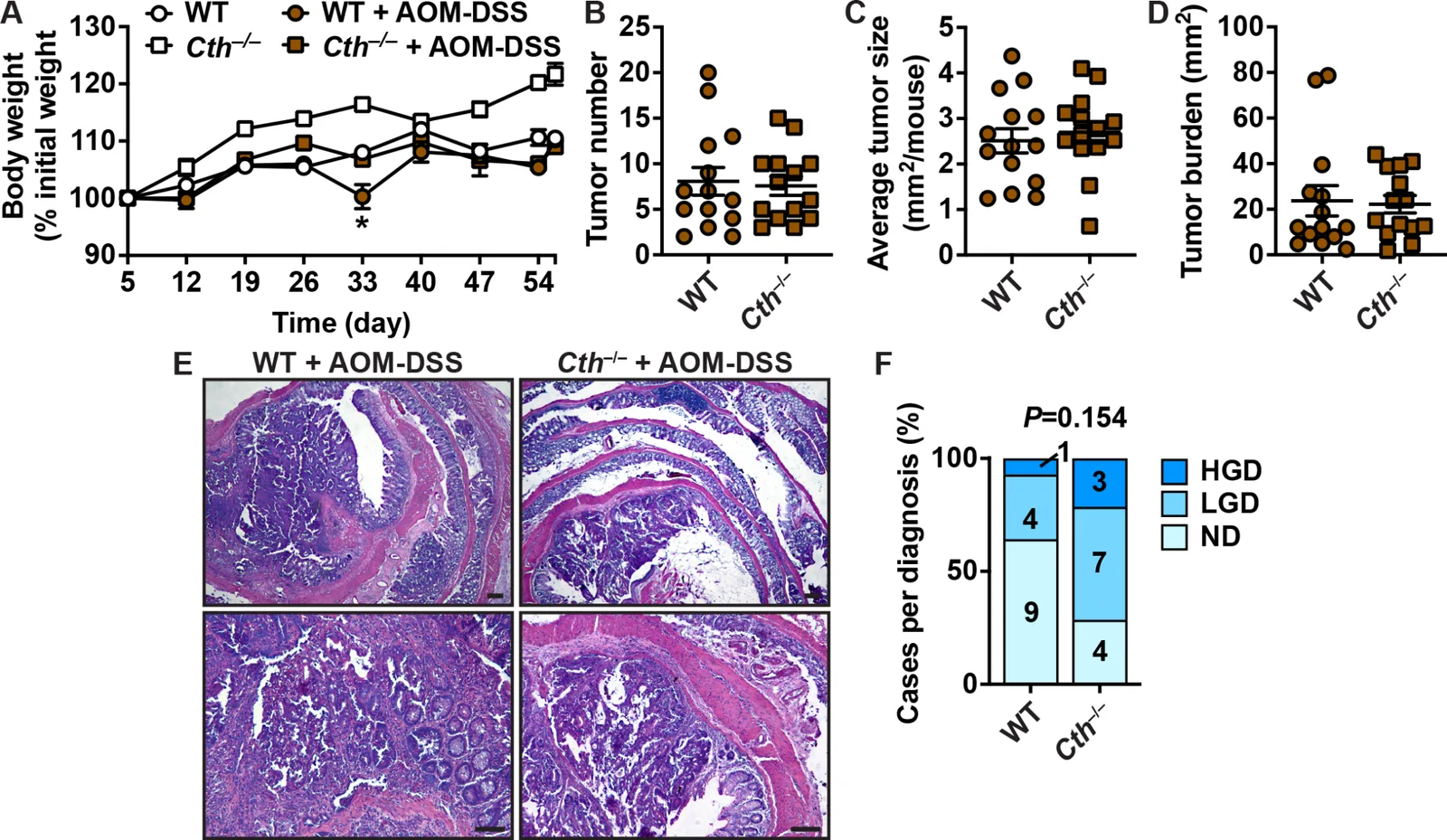
Effect of CTH on colon carcinogenesis. WT and *Cth*^–/–^ mice were treated or not with AOM-DSS. **A.** Body weights were measured weekly and are depicted as percentage of initial body weight. *P < 0.05 versus *Cth*^–/–^ + AOM-DSS. After 56 days, colons were removed and tumor number (**B**), tumor size (**C**), and tumor burden (**D**) were determined. **E.** Representative images of H&E staining; scale bars, 50 μm. **F.** Frequency of LGD and HGD; ND, no dysplasia. *P* was determined by Chi-square. *n* = 5 and *n* = 14 AOM-DSS-treated mice for both genotypes

Because *Cth* deletion protected from DSS colitis, we reasoned that it could have also a protective effect on development of CAC. At the second cycle of DSS treatment, we found that *Cth*^–/–^ mice lost significantly less weight than WT mice (Fig. 3A), as in the acute DSS model. However, we found that the number (Fig. 3B) and the size (Fig. 3C) of tumors, as well as the total tumor burden per colon (Fig. 3D) was similar in WT and *Cth*^–/–^ mice. Histologic assessment of H&E staining (Fig. 3E) demonstrated that the development of dysplasia was not different between the WT and *Cth*^–/–^ genotypes (Fig. 3F).

### Loss of the cystine transporter does not influence DSS colitis

One main physiological role of CTH is to generate cysteine from cystathionine, which is supported by our discovery that DSS-treated *Cth*^–/–^ mice have less cysteine in their colon compared to WT mice (Fig. 1C). Therefore, one mechanism by which *Cth* deletion could ameliorate DSS colitis might be by reducing intracellular cysteine. To mimic a context of intracellular cysteine deprivation, we used mice lacking the transporter SLC7A11 (Sato et al. 2005). This protein imports the anionic form of cysteine in exchange for glutamate and cells from *Slc7a11*^–/–^ mice have reduced intracellular cysteine (Sato et al. 2005).

**Fig. 4:**
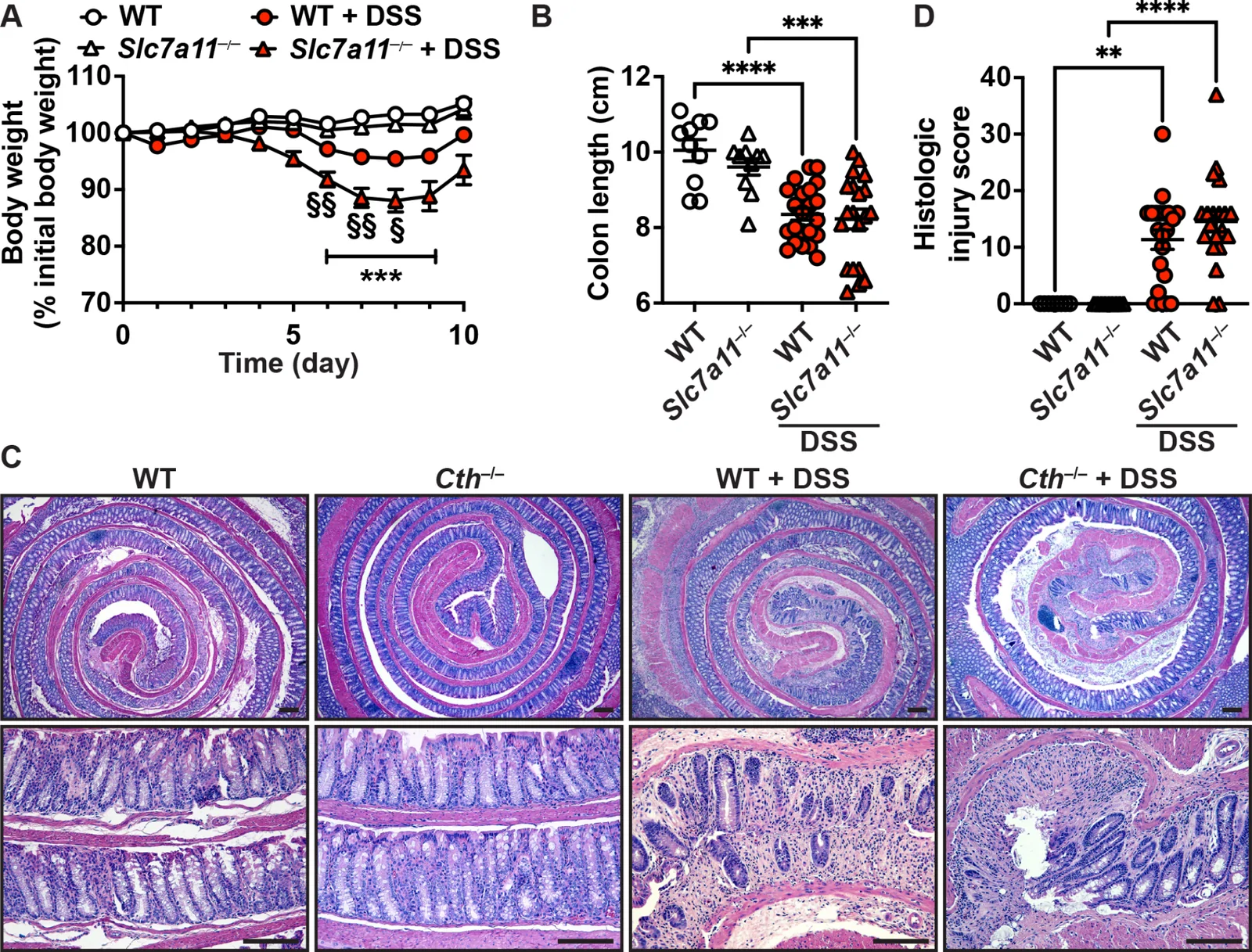
Colitis in *Slc7a11* mice. WT and *Slc7a11*^–/–^ mice were treated or not with 4% DSS for 5 days and then kept for 5 more days. (**A**) Body weights were assessed daily; §*P* < 0.05, §§*P* < 0.01 compared to WT + DSS. (**B**) The length of the colon was measured. **C-D.** H&E staining of the colons (**C**) was used to score histologic injury (**D**); scale bars, 100 μm. In **B** and **D**, **P < 0.01, ***P < 0.001, ****P < 0.0001 by one-way ANOVA and Tukey test. *n* = 10 and *n* = 22 DSS-treated mice for both genotypes

We first observed that *Slc7a11*^–/–^ mice lost significantly more weight than WT mice upon DSS treatment between day 6 and 8 (Fig. 4A). However, colon length was not affected by *Slc7a11* deletion (Fig. 4B). Further, we did not find a difference in histologic injury between WT and *Slc7a11*^–/–^ mice that were treated with DSS (Fig. 4C and D).

### CTH regulates the composition of the intestinal microbiota

**Fig. 5:**
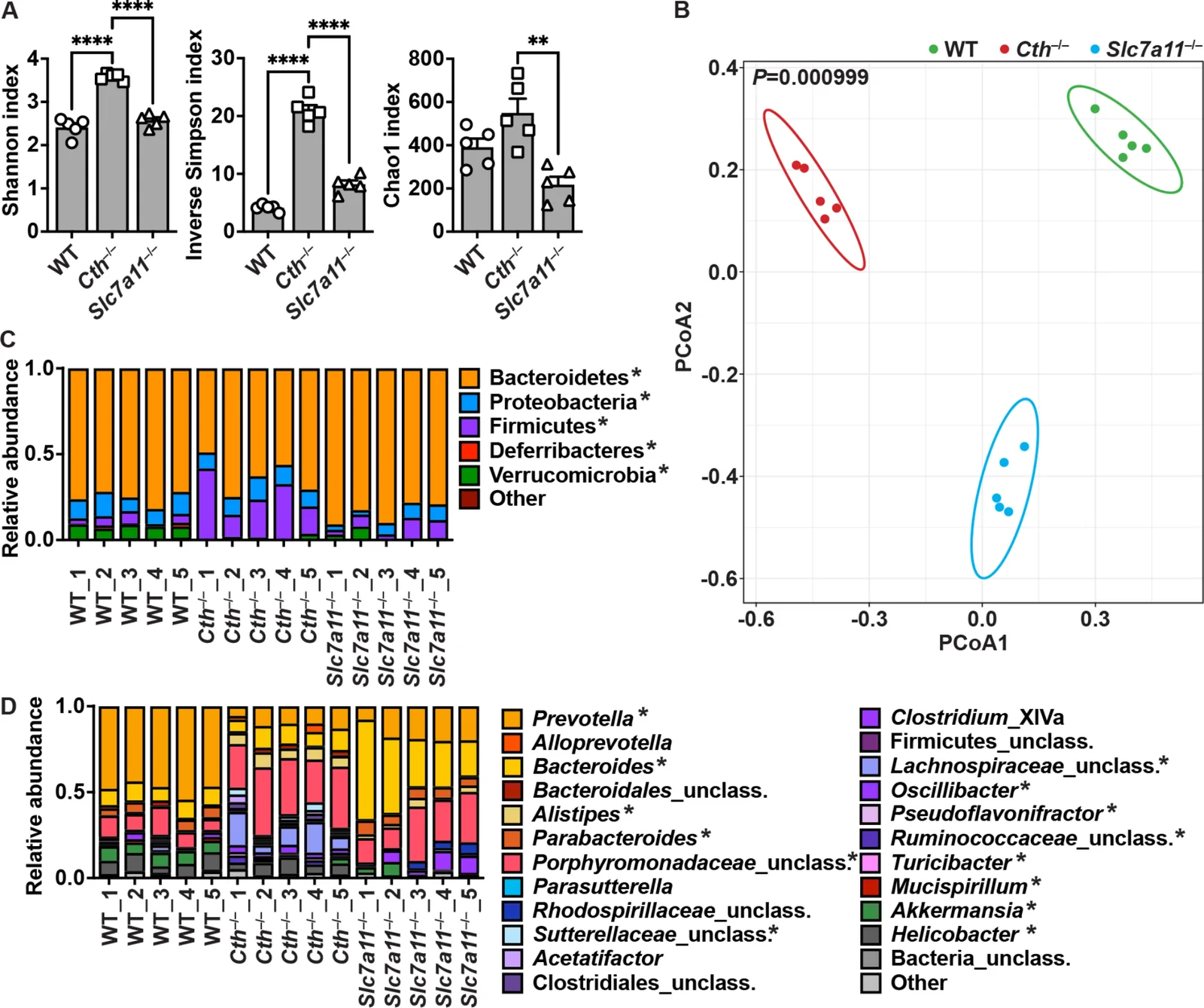
Composition of the gut microbiota. DNA was extracted from colonic feces of WT, *Cth*^–/–^, and *Slc7a11*^–/–^ mice. **A**. Alpha diversity, evaluated by the Shannon index, Inverse Simpson index, and Chao1 index; **P < 0.01, ****P < 0.0001 by one-way ANOVA and Tukey test. **B.** PCoA plot based on the unweighted UniFrac metric; *P* was determined by PERMANOVA. **C-D.** Bacterial community composition at phylum (**C**) and genus (**D**) levels, expressed as a ratio to the total community. *P < 0.05 by PERMANOVA

We then analyzed the composition of the intestinal microbiota, which can also regulate the development of colitis, in naïve WT, *Cth*^–/–^ and *Slc7a11*^–/–^ mice. Bacterial community diversity, assessed by Shannon and Inverse Simpson indexes (Fig. 5A), was significantly increased in *Cth*-deficient mice compared to both WT and *Slc7a11*^–/–^ mice. The Chao1 index, which is an indicator of total richness, was modestly increased in *Cth*^–/–^ mice compared to WT animals, although this did not reach statistical significance. We found that richness was reduced in *Slc7a11*-deficient mice compared to *Cth*^–/–^ mice (Fig. 5A). Thus, although the total number of bacterial species is similar in WT and *Cth*^–/–^ mice, the *Cth*-deficient animals exhibit a more diverse gut microbiota. According to the principal coordinate analysis (PcoA) based on the weighted UniFrac distance, the gut microbiota was clustered into three groups based on the genotype of the mice (Fig. 5B). The fecal microbiota of WT C57BL/6 mice and *Slc7a11*^–/–^ mice was mainly dominated by Bacteroidetes (Fig. 5C). We observed a significant reduction of this phylum in *Cth*-deficient mice that was accompanied by a significant increase of bacteria from the Firmicutes phylum (Fig. 5C). Although they were a minority, the phyla Deferribacteres and Verrumicomicrobia were less represented in *Cth*^–/–^ and *Slc7a11*^–/–^ mice compared to WT animals (Fig. 5C). *Prevotella*, *Porphyromonadaceae*, and *Bacteroides* were the prevalent genera in WT, *Cth*^–/–^, and *Slc7a11*^–/–^ mice, respectively (Fig. 5D). The *Lachnospiraceae* genus, which belongs to the Firmicutes phylum, was significantly increased in mice lacking *Cth* compared to WT and *Slc7a11*^–/–^ mice (Fig. 5D). There was also a marked reduction of the abundance of *Akkermansia* in *Cth*^–/–^ mice (Fig. 5D).

## Discussion

CTH is a major enzyme of the RTP that contributes to cysteine and H_2_S synthesis and, consequently, to sulfur metabolism and redox regulation. We observed that CTH is induced in the colon of mice with DSS colitis, as previously observed in rats (Flannigan et al. 2013). We also found herein that *Cth*-deficient mice exhibit less DSS colitis than WT mice, demonstrating that CTH supports colon inflammation. This does not appear to occur through the regulation of the mucosal concentration of cysteine, since mice lacking the cystine transporter SLC7A11 were not protected from colitis. However, the intestinal microbiota of *Cth*^–/–^ mice has increased diversity compared to WT and *Slc7a11*^–/–^ mice, suggesting that the gut microbial community is shaped by the host RTP. Interestingly, we also showed that *Cth* deletion does not affect tumorigenesis in a murine model of CAC. Therefore, we contend that CTH is a critical contributor to intestinal inflammation, but does not play a role in inflammation-induced neoplastic transformation.

In this report, we observed that *Cth* deletion ameliorates DSS colitis, assessed by body weight loss, colon length, and, more importantly, histological injury score; this overall score includes inflammatory parameters, epithelial injury, and their extent (Gobert et al. 2018, 2022, 2023). Accordingly, we found that the expression of the genes encoding for IL-1b and IL-17, two major cytokines that play a pathogenic role in colitis (Coccia et al. 2012; Ito et al. 2008), was also reduced in the colonic mucosa of *Cth*^–/–^ mice. We also confirmed that IL-1b protein was upregulated in DSS colitis and attenuated in *Cth*^–/–^ mice, which we demonstrated by both ELISA and IHC. Altogether, these data evidence that CTH supports the development of colitis in our DSS model. Supporting the contention that this enzyme can mediate inflammation in the gastrointestinal tract, we recently reported that *Cth* deletion dampens macrophage and T cell activation in the stomach of *H. pylori*-infected mice, leading to a reduction of gastritis (Latour et al. 2022). In addition, human and murine T cell activation and proliferation are reduced by silencing CTH and/or CBS (Miller et al. 2012). However, Akahoshi et al. have shown that C57BL/6 mice lacking *Cth* or *Mpst*, which encodes for the H_2_S generating enzyme mercaptopyruvate sulfurtransferase (THTM), do not show significant alteration of trinitrobenzene sulfonic acid (TNBS)- and oxazolone-induced colitis (Akahoshi et al. 2023). In contrast, the deleterious effect of *Cth* deletion on the pro-inflammatory response, including NLRP3 inflammasome activation and innate cytokine production, has been also reported (Qin et al. 2019); unfortunately, histological colitis was not investigated in this study (Qin et al. 2019). Further, Thanki et al. have reported that clinical and histological parameters are worsened in *Cth*^–/–^ mice, but these authors used a colitis score developed for ulcerative colitis (UC) patients (Geboes et al. 2000), which does not integrate epithelial injury nor extent of inflammation and damage (Thanki et al. 2022); moreover, these experiments were performed in mice with a C57BL/J6;129SvEv mixed background treated with 3% DSS (Thanki et al. 2022). Discrepancies between these studies and ours could be attributable to the differences of experimental procedures. It is also highly probable that the intestinal microbiota of the mice that have been used in these different studies differs due to dissimilar genetic backgrounds and animal facilities.

Because we found that *Cth*^–/–^ mice were protected from DSS colitis, we reasoned that this gene deletion could also influence the development of CAC induced by AOM and DSS. Surprisingly, we found that tumorigenesis and severity of dysplasia were identical in WT mice and those lacking CTH. Similarly, WT and *Cth*^–/–^ C57BL/J6;129SvEv mice treated with AOM-DSS displayed similar tumor burden after 80 days (Thanki et al. 2022). These data suggest that CTH is not a major contributor to carcinogenesis and tumor expansion in the inflamed colon. Nonetheless, it would be interesting to test the role of this enzyme in sporadic colorectal cancer (CRC), notably because *CTH i*) is stimulated by the WNT pathway in human CRC cells (Fan et al. 2014) and *ii*) is more highly expressed in human colonoids that harbor mutations in the genes implicated in CRC development, i.e., *APC* and *TP53*, *TP53* and *SMAD4*, or *TP53*, *SMAD4*, and *KRAS* (Ascencao et al. 2022). The CTH/H_2_S pathway results in proliferation and migration of CRC cells (Ascencao et al. 2022; Fan et al. 2014; Szabo et al. 2013), and growth of tumor xenografts in nude mice (Fan et al. 2014).

The activity of CTH has a major impact on amino acid metabolism and contributes to generation of numerous metabolites. In this context, it is probable that the deleterious effect of CTH on DSS colitis that we have observed has a multifactorial etiology. First, CTH generates cysteine and we observed that *Cth*^–/–^ mice have less cysteine in their colon compared to WT mice. Therefore, we reasoned that one mechanism by which CTH could support DSS colitis might be by reducing intracellular cysteine. Because cysteine is an antioxidant, the general concept is that this amino acid and its derivatives protect from inflammation. However, controversial results have emerged from a recent study showing that treatment with N-acetylcysteine increases the level of malondialdehyde, a reactive electrophile that we have implicated in colitis and CAC (Gobert et al. 2023), in the colon of mice with DSS colitis (da Paz Martins et al. 2023). To mimic a context of intracellular cysteine deprivation, we used mice lacking the cystine importer SLC7A11 (Sato et al. 2005). Note that *de novo* synthesis of cysteine and/or its import via other transporters cannot compensate for the need for intracellular cysteine in cells lacking SLC7A11 (Guo et al. 2023; Jyotsana et al. 2022). We found that DSS colitis was similar between WT and *Slc7a11*^–/–^ mice, suggesting that the deleterious effect of CTH in our model occurs independently of intracellular cysteine level.

Second, H_2_S is a gasotransmitter with anti-inflammatory properties synthesized by CTH and CBS during the RTP. Rectal injection of the H_2_S donor, NaHS, protects Wistar rats from dinitrobenzene sulfonic acid colitis (Goyal et al. 2015) and daily intraperitoneal injections dampens DSS colitis in C57BL/6 mice (Qin et al. 2019); in this last study, the treatment with NaHS was not investigated in *Cth*-deficient mice, which exhibited increased colitis (Qin et al. 2019). Thus, these data demonstrate that exogenous H_2_S might be protective in the colon, but do not directly establish that CTH exerts a protective effect through H_2_S. In contrast, excess H_2_S production has been suggested to be involved in the pathogenesis of IBD (Mottawea et al. 2016; Rowan et al. 2009). Fecal sulfide concentration and production were found to be elevated in patients with UC (Pitcher et al. 2000; Levine et al. 1998). Thus, H_2_S may cause mucus disruption by reducing its disulfide bonds (Ijssennagger et al. 2016), and the administration of the H_2_S-producing bacterium *Desulfovibrio indonesiensis* and *Atopobium parvulum* exacerbates the development of TNBS colitis (Figliuolo et al. 2017) and spontaneous colitis in *Il10*^−/−^ mice (Mottawea et al. 2016), respectively. Interestingly, we observed that WT mice with DSS colitis have increased CTH expression in their colon, which can in turn lead to increased H_2_S generation. In this context, *Cth* deletion could lead to the amelioration of DSS colitis by reducing endogenous H_2_S generation. However, it should be recognized that the cysteine aminotransferase/THTM metabolic pathway is a major source of colonic H_2_S compared to the RTP in healthy and inflamed colon (Flannigan et al. 2013).

Third, we investigated whether CTH can modulate the composition of the intestinal microbiota, which can play a major role in the pathophysiology of intestinal inflammation (Lee and Chang 2021). First, we found an overall increased diversity and richness in the fecal microbiota of *Cth*^–/–^ mice compared to both WT and *Slc7a11*^–/–^ mice, although the b-diversity analysis indicated that bacterial communities of the three genotypes are dissimilar. Loss of diversity is mainly associated with development of more severe colitis in mice (Singh et al. 2019) and is a hallmark of IBD pathogenesis (Frank et al., 2007; Willing et al., 2010), suggesting that CTH may support colon inflammation by reducing diversity. Further, the taxonomic distribution of the bacteria in each genotype indicated that the fecal microbiota of C57BL/6 mice, as we previously reported (Gobert et al. 2022, 2023; Singh et al. 2019), and *Slc7a11*^–/–^ mice was mainly dominated by Bacteroidetes; within this phylum, *Prevotella* and *Bacteroides* were the prevalent genera in WT and *Slc7a11*^–/–^ mice, respectively. *Cth* deficiency was associated with a reduction of Bacteroidetes and an increase of Firmicutes. At the genus level, *Porphyromonadaceae* (Bacteroidetes) and *Lachnospiraceae* (Firmicutes), which are associated with the resistance of mice to infectious colitis (Ferreira et al. 2011), were dominant in *Cth*^–/–^ mice. Interestingly, the prevalence of Bacteroidetes is often increased and Firmicutes reduced in patients with IBD (Santana et al. 2022), leading to the concept that a decrease of the Firmicutes/Bacteroidetes ratio is considered as a dysbiosis and potentially contributes to the etiology/progression of colitis (Stojanov et al. 2020). Our present data showed an increased Firmicutes/Bacteroidetes ratio in *Cth*^–/–^ mice, which are protected from DSS colitis, suggesting that CTH activity in the colon may sustain a pro-inflammatory microbiota through the dysbiosis of this ratio. In this global context, the mechanism by which host CTH activity affects the ecology of the microbiological intestinal community remains to be elucidated.

The RTP is essential for homeostasis. However, we found that dysregulation of this metabolic pathway, notably during infection, results in increased pathology (Latour et al. 2022). The present paper shows that a similar concept occurs for colitis. Further investigations are warranted to define the molecular/cellular mechanism by which CTH supports colonic inflammation. However, we present herein the first line of evidence that the host RTP regulates the composition of the intestinal microbiota as a potential causal factor in colitis.

## Data Availability

The 16 S rRNA sequencing of the fecal microbiota has been deposited on the Sequence Read Archive website with the BioProject ID PRJNA1096754.
